# Supplementary material for: The impact of long-term conditions on the progression of frailty
Source: PLoS One. 2023 Apr 6;18(4):e0284011. doi: 10.1371/journal.pone.0284011 (PMC10079116; doi:10.1371/journal.pone.0284011)
Supplement: S1 Text — (DOCX) [file pone.0284011.s001.docx]

| 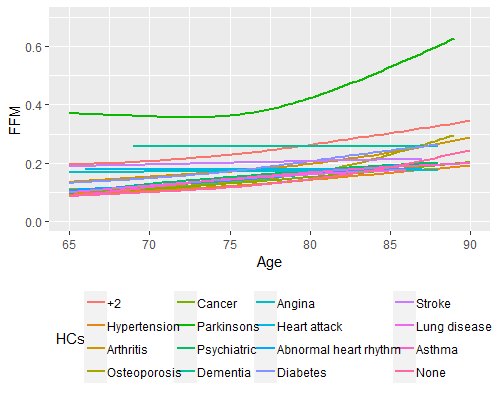  Figure 1: FMM vs age grouped by LTCs (Females) |
| --- |
|  |
| 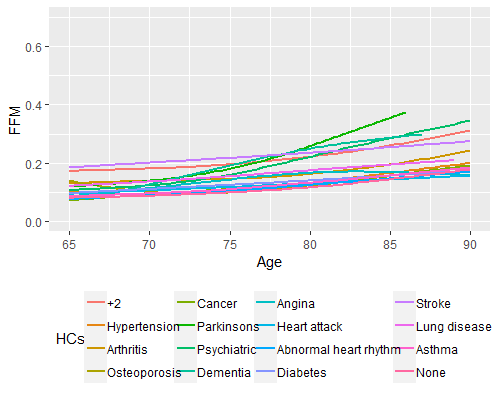  Figure 2: FMM vs age grouped by LTCs (Males) |
| Congestive heart failure and Alzheimer's have been omitted from both figures above since they are small observations. |

Table 1: The mean of the FMM for females with three age categories grouped by: two or more health conditions (HC), a specific one HC or none HC.

|  |  | Age | | |
| --- | --- | --- | --- | --- |
|  |  | 65-74 | 57-84 | 85-90 |
| Group | | FFM | FFM | FFM |
| multimorbidity (2^+^) | | 0.208 | 0.262 | 0.325 |
| One health condition | Hypertension | 0.105 | 0.141 | 0.178 |
|  | Arthritis | 0.151 | 0.193 | 0.274 |
|  | Osteoporosis | 0.117 | 0.166 | 0.283 |
|  | Cancer | 0.109 | 0.154 | 0.195 |
|  | Parkinson’s | 0.361 | 0.421 | 0.722 |
|  | Psychiatric | 0.120 | 0.179 | 0.173 |
|  | Dementia | 0.282 | 0.194 | 0.409 |
|  | Angina | 0.163 | 0.173 | 0.265 |
|  | Heart attack | 0.176 | 0.190 | 0.060 |
|  | Abnormal heart rhythm | 0.121 | 0.166 | 0.117 |
|  | Diabetes | 0.139 | 0.215 | 0.250 |
|  | Stroke | 0.179 | 0.223 | 0.326 |
|  | Lung disease | 0.117 | 0.176 | 0.131 |
|  | Asthma | 0.111 | 0.160 | 0.192 |
| None | | 0.097 | 0.143 | 0.219 |

Table 2: The mean of the FMM for males with three age categories grouped by: two or more health conditions (HC), a specific one HC or none HC.

|  |  | 65-74 | 57-84 | 85-90 |
| --- | --- | --- | --- | --- |
| Group | | FFM | FFM | FFM |
| multimorbidity (2^+^) | | 0.182 | 0.221 | 0.289 |
| One health condition | Hypertension | 0.098 | 0.129 | 0.181 |
|  | Arthritis | 0.138 | 0.161 | 0.227 |
|  | Osteoporosis | 0.106 | 0.125 | 0.242 |
|  | Cancer | 0.093 | 0.121 | 0.196 |
|  | Parkinson’s | 0.143 | 0.244 | 0.467 |
|  | Psychiatric | 0.119 | 0.247 | 0.366 |
|  | Alzheimer | 0.359 | 0.363 | 0.336 |
|  | Dementia | 0.104 | 0.270 | 0.186 |
|  | Angina | 0.112 | 0.174 | 0.119 |
|  | Heart attack | 0.101 | 0.136 | 0.165 |
|  | Congestive heart failure | 0.105 | --- | --- |
|  | Abnormal heart rhythm | 0.086 | 0.124 | 0.163 |
|  | Diabetes | 0.108 | 0.138 | 0.207 |
|  | Stroke | 0.206 | 0.208 | 0.361 |
|  | Lung disease | 0.131 | 0.188 | 0.187 |
|  | Asthma | 0.091 | 0.142 | 0.171 |
| None | | 0.086 | 0.116 | 0.161 |

In table 1, two health condition were omitted, which were congestive heart failure and Alzheimer's, because there were not females participants have these conditions separately.

Table 3: 62 deficits for frailty index

| Domain |  | Item | Domain |  | Item |
| --- | --- | --- | --- | --- | --- |
| Mobility1 | 1 | difficulty walking 100 yards | Heart problems | 32 | high blood pressure or hypertension |
|  | 2 | difficulty sitting 2 hours |  | 33 | Angina |
|  | 3 | difficulty getting up from a chair after sitting long periods |  | 34 | heart attack |
|  | 4 | difficulty climbing several flights of stairs without resting |  | 35 | congestive heart failure |
|  | 5 | difficulty climbing one flight stairs without resting |  | 36 | abnormal heart rhythm |
|  | 6 | difficulty stooping, kneeling or crouching |  | 37 | diabetes or high blood sugar |
|  | 7 | difficulty reaching or extending arms above shoulder level |  | 38 | Stroke |
|  | 8 | difficulty pulling or pushing large objects | chronic disease | 39 | lung disease |
|  | 9 | difficulty lifting or carrying weights over 10 pounds |  | 40 | Asthma |
|  | 10 | difficulty picking up 5p coin from the table |  | 41 | Arthritis |
| Mobility 2 | 11 | difficulty dressing, including putting on shoes and socks |  | 42 | Osteoporosis |
|  | 12 | difficulty walking across a room |  | 43 | Cancer |
|  | 13 | difficulty bathing or showering |  | 44 | Parkinsons |
|  | 14 | difficulty eating, such as cutting up food |  | 45 | Psychiatric |
|  | 15 | difficulty getting in and out of bed |  | 46 | Alzheimer's |
|  | 16 | difficulty using the toilet, including getting up or down |  | 47 | Dementia |
|  | 17 | difficulty using a map to figure out how to get around a strange place | self-reported and operation | 48 | Self-reported eyesight |
|  | 18 | preparing a hot meal |  | 49 | Self-reported general health |
|  | 19 | shopping for groceries |  | 50 | Self-reported hearing |
|  | 20 | making telephone calls |  | 51 | Fallen down |
|  | 21 | taking medications |  | 52 | fractured hip |
|  | 22 | doing work around the house or garden |  | 53 | had joint replacement |
|  | 23 | managing money, such as bills and expenses |  | 54 | had pain whilst walking |
| Psychology | 24 | Whether felt depressed much of the time during the past week | memory test | 55 | correct day of month given |
|  | 25 | Whether felt everything they did during the past week was an effort |  | 56 | correct month given |
|  | 26 | felt their sleep was restless during the past week |  | 57 | correct year given |
|  | 27 | Whether was happy much of the time during the past week |  | 58 | correct day given |
|  | 28 | Whether felt lonely much of the time during the past week |  | 59 | ~~prompt given for prospective memory test~~ |
|  | 29 | Whether enjoyed life much of the time during the past week |  | 60 | Number of words recalled immediately |
|  | 30 | Whether felt sad much of the time during the past week |  | 61 | Number of animals mentioned |
|  | 31 | Whether could not get going much of the time during the past week |  | 62 | Number of words recalled after a delay |

Table 4: A discrete time model (logistic regression with cloglog link function)

| Female | | | | | |
| --- | --- | --- | --- | --- | --- |
|  | β | se | Exp(β) | Z_value | p |
| (Intercept) | -4.115 | 0.202 | 0.016 | -20.345 | 0 |
| I(Age - 70) | 0.071 | 0.006 | 1.073 | 12.77 | 0 |
| Scale FFM | 0.461 | 0.041 | 1.586 | 11.305 | 0 |
| HC=1 | -0.123 | 0.223 | 0.885 | -0.551 | 0.581 |
| HC=2^+^ | 0.003 | 0.201 | 1.003 | 0.016 | 0.987 |
| time2 | -0.381 | 0.16 | 0.683 | -2.379 | 0.017 |
| time3 | -0.367 | 0.16 | 0.693 | -2.294 | 0.022 |
| time4 | -0.337 | 0.154 | 0.714 | -2.189 | 0.029 |
| time5 | -0.128 | 0.145 | 0.88 | -0.886 | 0.376 |
| time6 | -17.159 | 291.243 | 0 | -0.059 | 0.953 |
| Male | | | | | |
|  | β | se | Exp(β) | Z_value | p |
| (Intercept) | -3.86 | 0.20 | 0.02 | -19.42 | 0.00 |
| I(Age - 70) | 0.06 | 0.01 | 1.07 | 12.35 | 0.00 |
| Scale FFM | 0.53 | 0.04 | 1.69 | 13.95 | 0.00 |
| HC=1 | 0.48 | 0.21 | 1.62 | 2.27 | 0.02 |
| HC=2^+^ | 0.68 | 0.20 | 1.98 | 3.43 | 0.00 |
| time2 | -0.43 | 0.14 | 0.65 | -3.05 | 0.00 |
| time3 | -0.29 | 0.14 | 0.75 | -2.17 | 0.03 |
| time4 | -0.41 | 0.13 | 0.66 | -3.05 | 0.00 |
| time5 | -0.73 | 0.14 | 0.48 | -5.05 | 0.00 |
| time6 | -16.70 | 192.27 | 0.00 | -0.09 | 0.93 |

We used the logistic regression model as a discrete-time model with *clogclog* link function rather than cox regression due the time points were registered in two years (interval-censored). We only used the first six waves because the death were reported until wave 6.

In table 4, we presented the results for the logistic regression model. We notice that the FFM in female and male models was considered a strong predictor for mortality; HR 1.59 and 1.69, respectively. Both models tested proportional hazards by interacting time and long-term health conditions. There is no sign of non-proportionality for all covariates in both models except the FFM in the male model.

Table 5: secondary exposure distribution over the nine waves

| wave | 1 | 2 | 3 | 4 | 5 | 6 | 7 | 8 | 9 |
| --- | --- | --- | --- | --- | --- | --- | --- | --- | --- |
| N | 5455 | 4638 | 4308 | 5041 | 5252 | 5528 | 5434 | 5346 | 5300 |
| **Age** (mean (SD)) | 73.79 (6.35) | 73.97 (6.33) | 74.36 (6.50) | 73.71 (6.33) | 73.91 (6.50) | 73.65 (6.55) | 73.73 (6.48) | 73.88 (6.47) | 74.15 (6.56) |
| **Male** | 2440 (44.7) | 2071 (44.7) | 1914 (44.4) | 2315 (45.9) | 2408 (45.8) | 2577 (46.6) | 2500 (46.0) | 2478 (46.4) | 2406 (45.4) |
| **education (%)** |  |  |  |  |  |  |  |  |  |
| High | 849 (15.6) | 857 (18.5) | 1026 (23.8) | 1330 (26.4) | 1504 (28.6) | 1525 (27.6) | 1568 (28.9) | 1577 (29.5) | 1984 (37.4) |
| Med or foreign | 1645 (30.2) | 1522 (32.8) | 1509 (35.0) | 1779 (35.3) | 1942 (37.0) | 2140 (38.7) | 2237 (41.2) | 2262 (42.3) | 2141 (40.4) |
| Low | 2944 (54.0) | 2248 (48.5) | 1758 (40.8) | 1900 (37.7) | 1772 (33.7) | 1772 (32.1) | 1546 (28.5) | 1386 (25.9) | 1138 (21.5) |
| NA | 17 ( 0.3) | 11 ( 0.2) | 15 ( 0.3) | 32 ( 0.6) | 34 ( 0.6) | 91 ( 1.6) | 83 ( 1.5) | 121 ( 2.3) | 37 ( 0.7) |
| **Wealth class (%)** |  |  |  |  |  |  |  |  |  |
| Richest | 924 (16.9) | 761 (16.4) | 701 (16.3) | 827 (16.4) | 918 (17.5) | 1008 (18.2) | 1026 (18.9) | 992 (18.6) | 1089 (20.5) |
| Rich | 991 (18.2) | 894 (19.3) | 848 (19.7) | 986 (19.6) | 986 (18.8) | 1095 (19.8) | 1110 (20.4) | 1069 (20.0) | 1110 (20.9) |
| Average | 1141 (20.9) | 1014 (21.9) | 933 (21.7) | 1101 (21.8) | 1135 (21.6) | 1183 (21.4) | 1173 (21.6) | 1161 (21.7) | 1124 (21.2) |
| Poor | 1341 (24.6) | 1089 (23.5) | 1060 (24.6) | 1210 (24.0) | 1256 (23.9) | 1278 (23.1) | 1166 (21.5) | 1145 (21.4) | 1094 (20.6) |
| Poorest | 989 (18.1) | 840 (18.1) | 663 (15.4) | 786 (15.6) | 841 (16.0) | 859 (15.5) | 850 (15.6) | 911 (17.0) | 804 (15.2) |
| NA | 69 ( 1.3) | 40 ( 0.9) | 103 ( 2.4) | 131 ( 2.6) | 116 ( 2.2) | 105 ( 1.9) | 109 ( 2.0) | 68 ( 1.3) | 79 ( 1.5) |

Table 6: The years of Collecting ELSA data and the mean of CPI per two years.

| Wave | year | price index | mean PI | mean PI/100 |
| --- | --- | --- | --- | --- |
| 1 | 2002 | 75.7 | 76.2 | 0.762 |
|  | 2003 | 76.7 |  |  |
| 2 | 2004 | 77.8 | 78.6 | 0.786 |
|  | 2005 | 79.4 |  |  |
| 3 | 2006 | 81.4 | 82.35 | 0.8235 |
|  | 2007 | 83.3 |  |  |
| 4 | 2008 | 86.2 | 87.05 | 0.8705 |
|  | 2009 | 87.9 |  |  |
| 5 | 2010 | 90.1 | 91.85 | 0.9185 |
|  | 2011 | 93.6 |  |  |
| 6 | 2012 | 96 | 97.1 | 0.971 |
|  | 2013 | 98.2 |  |  |
| 7 | 2014 | 99.6 | 99.8 | 0.998 |
|  | 2015 | 100 |  |  |
| 8 | 2016 | 101 | 102.3 | 1.023 |
|  | 2017 | 103.6 |  |  |
| 9 | 2018 | 106 | 106.9 | 1.069 |
|  | 2019 | 107.8 |  |  |

| 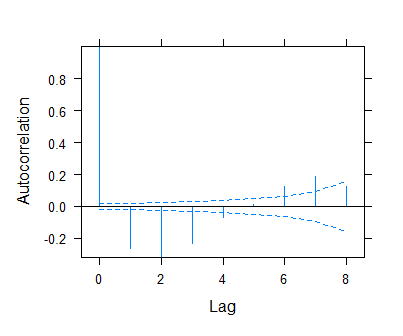 | 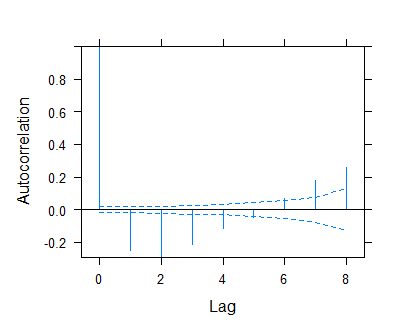 |
| --- | --- |
| 1. ACF plot for Female model | 1. ACF plot for Female model |
| Figure 3: ACF for males and females model | |

Table 7: Multilevel growth model for males and females (only complete cases)

|  | Male | | | | | | Female | | | | | |
| --- | --- | --- | --- | --- | --- | --- | --- | --- | --- | --- | --- | --- |
|  | Unadjusted model | | | Adjusted model | | | Unadjusted model | | | Adjusted model | | |
| **fixed effects** | β | RSE |  | β | RSE |  | β | RSE |  | β | RSE |  |
| (Intercept) | 0.15* | 0.002 |  | 0.11* | 0.003 |  | 0.19* | 0.002 |  | 0.13* | 0.004 |  |
| Age c(70)/10 |  |  |  | 0.03* | 0.003 |  |  |  |  | 0.04* | 0.004 |  |
| Age^2^ c(70)/10 |  |  |  | 0.01* | 0.004 |  |  |  |  | 0.01* | 0.005 |  |
| **Net wealth (ref. poor)** | | | | | | | | | | | | |
| Richest |  |  |  | -0.01* | 0.002 |  |  |  |  | -0.01* | 0.002 |  |
| Average |  |  |  | 0.02* | 0.002 |  |  |  |  | 0.02* | 0.002 |  |
| **Education (ref. low)** | | | | | | | | | | | | |
| High education |  |  |  | -0.01* | 0.003 |  |  |  |  | -0.01* | 0.003 |  |
| Meddle education |  |  |  | 0.03* | 0.003 |  |  |  |  | 0.03* | 0.003 |  |
| **Health condition (ref. HC=0)** |  | | | | | | | | | | | |
| HC (1) |  |  |  | 0.02* | 0.003 |  |  |  |  | 0.02* | 0.003 |  |
| HC (2^+^) |  |  |  | 0.06* | 0.003 |  |  |  |  | 0.06* | 0.004 |  |
| Age c(70)/10: HC (1) |  |  |  | 0.003 | 0.004 |  |  |  |  | -0.01 | 0.005 |  |
| Age^2^ c(70)/10: HC (1) |  |  |  | 0.001 | 0.005 |  |  |  |  | 0.002 | 0.006 |  |
| Age c(70)/10: HC (2^+^) |  |  |  | 0.02* | 0.004 |  |  |  |  | 0.001 | 0.005 |  |
| Age^2^ c(70)/10: HC (2^+^) |  |  |  | 0.01* | 0.005 |  |  |  |  | 0.01 | 0.005 |  |
| **Random effects** |  | lower | upper |  | lower | upper |  | lower | upper |  | lower | upper |
| Intercept (sd) | 0.10 | 0.10 | 0.10 | 0.09 | 0.07 | 0.11 | 0.11 | 0.11 | 0.12 | 0.10 | 0.10 | 0.10 |
| Age (sd) |  |  |  | 0.06 | 0.03 | 0.15 |  |  |  | 0.06 | 0.05 | 0.06 |
| Age^2^ (sd) |  |  |  | 0.04 | 0.01 | 0.20 |  |  |  | 0.04 | 0.03 | 0.05 |
| Error (sd) | 0.06 | 0.06 | 0.06 | 0.05 | 0.05 | 0.05 | 0.07 | 0.06 | 0.07 | 0.06 | 0.06 | 0.06 |
| **Model fit** |  | | | | | | | | | | | |
| AIC | -35668.49 | | | -38867.66 | | | -38830.52 | | | -42190.92 | | |

*p < 0.01
