# Supplementary material for: The impact of long-term conditions on the progression of frailty
Source: PLoS One. 2023 Apr 6;18(4):e0284011. doi: 10.1371/journal.pone.0284011 (PMC10079116; doi:10.1371/journal.pone.0284011)
Supplement: Attachment — Submitted filename: Response to Reviewers.docx [file pone.0284011.s002.docx]

Review 1: We added two limitations to the cumulative deficit model in the discussion section and defined long-term conditions (LTCs) in the introduction. However, the 16 clinical conditions were listed in the method section, and we believe it is appropriate to record them there.

Review 2: I changed the background of the figures in the manuscript and added the legends. But we did not do that for the supplementary file.

Regards,

Ali
